# Connectivity maps based analysis of EEG for the advanced diagnosis of schizophrenia attributes

**DOI:** 10.1371/journal.pone.0185852

**Published:** 2017-10-19

**Authors:** Zack Dvey-Aharon, Noa Fogelson, Abraham Peled, Nathan Intrator

**Affiliations:** 1 Blavatnik School of Computer Science, Tel-Aviv University, Tel-Aviv, Israel; 2 EEG and Cognition Laboratory, University of A Coruña, A Coruña, Spain; 3 Ruth and Bruce Rappaport Faculty of Medicine, Technion, Haifa, Israel; 4 Institute for Psychiatric Studies, Sha’ar Menashe Mental Health Center, Hadera, Israel; National University of Defense Technology College of Mechatronic Engineering and Automation, CHINA

## Abstract

This article presents a novel connectivity analysis method that is suitable for multi-node networks such as EEG, MEG or EcOG electrode recordings. Its diagnostic power and ability to interpret brain states in schizophrenia is demonstrated on a set of 50 subjects that constituted of 25 healthy and 25 diagnosed with schizophrenia and treated with medication. The method can also be used for the automatic detection of schizophrenia; it exhibits higher sensitivity than state-of-the-art methods with no false positives. The detection is based on an analysis from a minute long pattern-recognition computer task. Moreover, this connectivity analysis leads naturally to an optimal choice of electrodes and hence to highly statistically significant results that are based on data from only 3–5 electrodes. The method is general and can be used for the diagnosis of other psychiatric conditions, provided an appropriate computer task is devised.

## 1. Introduction

Electroencephalography (EEG) has become a preferred tool for general brain state interpretations, despite its low spatial resolution. The main alternative to the EEG for recording and analyzing brain activity is the MRI (or functional MRI), a high-resolution tool based on magnetic resonance. Although superior to the EEG in spatial resolution, fMRI-based recordings have poor temporal resolution (compared to EEG recordings), and the acquisition device is several thousand times more expensive. It can only be used in clinical settings, is sensitive to the subjects’ movement and cannot be used for continuous brain state monitoring. Thus, analyses based on EEG recordings offer significant advantages and enable many applications. Therefore, while analyses of network connectivity conducted to gain a broader knowledge of clinically relevant physiological attributes of mental disorders have largely been performed based on fMRI, using EEG data could be of tremendous use in medicine and research.

EEG data have primarily been regarded as complementary to fMRI data, **[1][2][3]**, but more recently, the EEG has been used as a tool for the early detection of changes in general cognitive activity, for example, in emotional states **[4][5]**, cognitive states **[6][7]**, analysis of epileptic seizures **[8][9]** and autism **[10][11]**. Other recent EEG applications have been in the area of brain-machine interfaces **[12]**.

EEG studies have suggested that changes in functional connectivity occur in schizophrenia patients **[13]**, and a significant difference in theta-frequency activity has been found, as well **[14]**. Recent studies suggest that a single- or few- electrode approach may be used to classify changes in schizophrenia **[15]** and that single trial-based analysis may be used to characterize emotional states **[16]**.

As connectivity research using fMRI has become common in analyzing mental pathologies **[11][17]** including resting state schizophrenia demonstrating strong discrimination **[18][19]**, it has inspired the study of EEG-based connectivity analysis. In particular, EEG enables finer temporal connectivity analysis.

Motivated by fMRI, multiple-electrode methodologies tend to look for the most relevant connections based on a dense configuration **[20]**. More recent work has attempted to examine all possible direct connections between electrodes to enable real-time clinical diagnosis **[21][22]**. A recent connectivity study of resting EEG networks successfully demonstrated the ability to discriminate between Psychogenic non-epileptic seizures (PNES) and Epilepsy **[23]**.

The present study is inspired by feature-connectivity research that is based solely on EEG recordings. We describe a novel connectivity analysis tool—Connectivity Maps—for the analysis of connectivity between nodes. The main difference between this method and conventional connectivity analysis **[22]** is that the connectivity between nodes is measured in terms of the connectivity between each node and a third, reference node.

In connectivity analysis, a total of *n*^2^ maps are created (*n* from each source electrode), and the largest changes between each pair of electrodes are determined. Thus, the properties obtained using such an approach enrich the current direct perception of connectivity.

This methodology is demonstrated using data from schizophrenia patients that has been examined previously **[24]**. Applying the proposed methodology to this data set improved the ability to discriminate between patients and healthy controls and provided insight into physiological aspects of this disease.

## 2 Methodology – A novel measure of connectivity

### 2.1 –Connectivity maps

The motivation for the proposed connectivity analysis is best explained by the following analysis of airline traffic. Consider the air traffic from BOS to ATL and BOS to DEN. A classical connectivity analysis of the nodes ATL and DEN would examine the connection between these two cities. For the sake of argument, let us assume that all the flights of a certain airline from BOS to DEN go through ATL. We consider a more refined, high dimensional notion of connectivity by analyzing the changes in connectivity between ATL and DEN with reference to flights that emanated from other locations, such as BOS. By observing that there was a reduction in connectivity between BOS and DEN but no change in or even an increase in connectivity between BOS and ATL. In other words, we may observe that when the flights of one airline company that flies from BOS to DEN via ATL are canceled, it is possible that those passengers book alternative flights with another carrier due to the cancellations. Note that the traffic between ATL and DEN includes flights by all other carriers; thus, it would be difficult to observe the reduction in traffic due to cancellations by a single carrier. By finding a city, BOS, from which a given carrier’s flights to DEN only go through ATL, a cancellation becomes more apparent.

**Fig 1:**
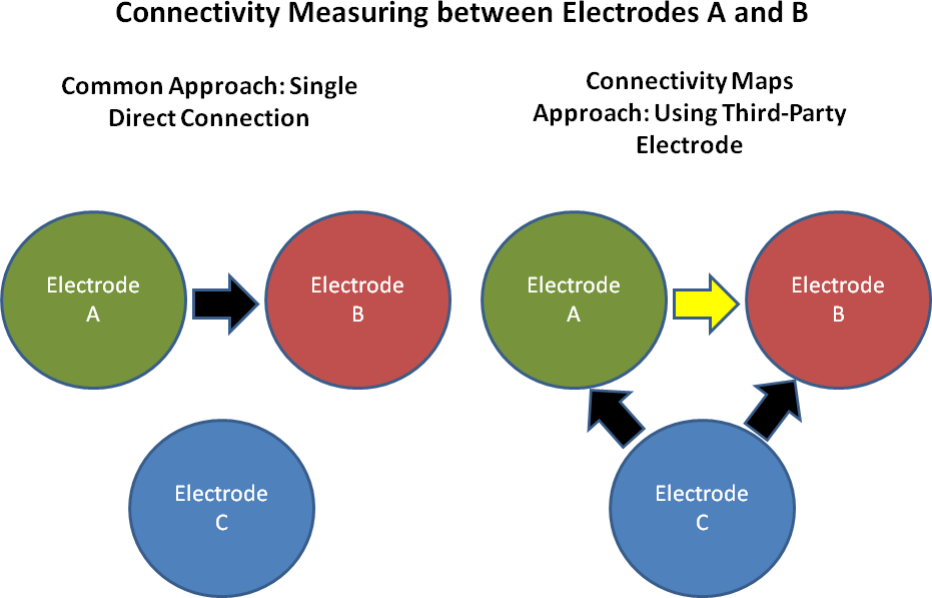
In addition to the direct connectivity approach (left panel), the connectivity maps approach brings features involving a reference electrode when addressing bi-electrode connectivity. Adding such an electrode expands the connectivity approach, as the relative changes in connectivity are uncovered.

In Electroencephalography, such technique may reveal the same type of significant insights, while the nodes in this case are simply the electrodes placed on the scalp and a reference-based connectivity function is required to measure the relationships between them. Therefore, in the same way, we analyze the connectivity between electrodes A and B (Fig 1) by examining either the direct connectivity or information transfer between A and B (left panel) or the induced change in connectivity between A and B, as seen by the change in the information transfer between reference electrode C and A with respect to the information transfer between C and B.

Our proposed discrimination and classification mechanism is based on a novel connectivity analysis tool that we term “Connectivity Maps”. These maps are generated in a relatively simple process that consists of the following 6 steps:

1. Several preprocessing tasks are performed, and the raw signals are broken into relevant intervals. Technical description of the preprocessing phase is detailed below under “Experimental Setup”.
2. The signal in several frequency bands is decomposed and reconstructed, and then time windows that maximize the correlation between electrodes are sought.
3. A network of correlations with respect to different base or reference electrodes is constructed.
4. Fisher-based feature extraction (See below definitions for Fisher and Relative Fisher matrices) from the set of connectivity maps obtained for the different reference electrodes is performed.
5. An optimization analysis is conducted based on the chronological presence of stimuli.
6. Post analysis and processing of the results is performed, including discrimination between the correlation matrix and Fisher-based features of the healthy subjects and those of the schizophrenia patients. In addition to the resulted parameters, this step is also performed under different constraints, such as specific time frames or following specific stimuli, for a broader statistical analysis.

**Fig 2:**
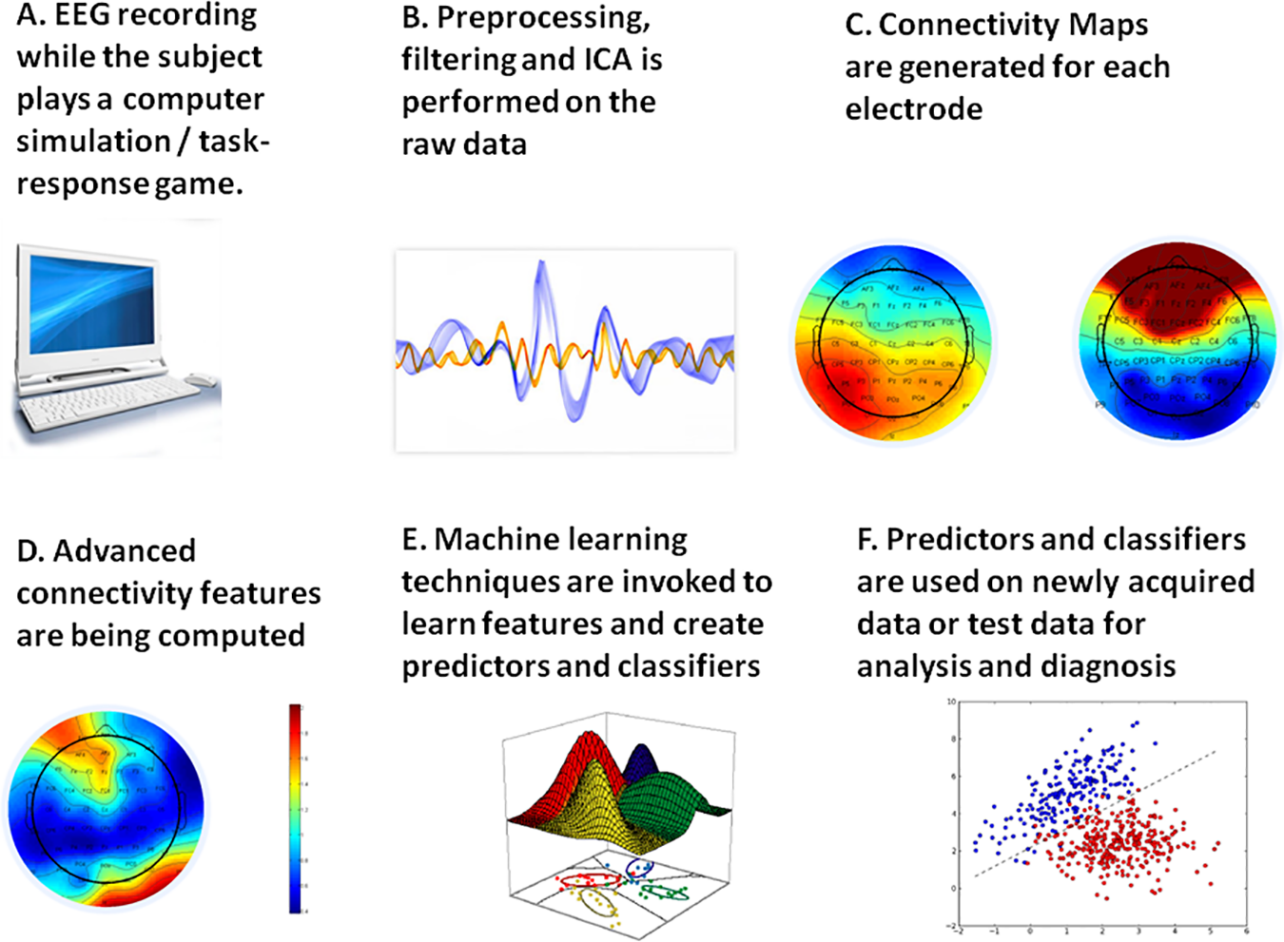
Phases of the connectivity maps methodology.

These steps are shown in Fig 2 below.

Given a window size W, a maximum phase *φ* and a number of trials *i* ∈ {1..*t*} for an electrode, let us define a connectivity function *F*_*conn*_ between two electrodes, such as a cross correlation, as

$$F_{Conn}(w)=F_{CrossCor}(A,B)=argmax_{\varphi }CrossCor_{w,\varphi }(avg(A_{i}),avg(B_{i}))$$

$$F_{Conn}\equiv subjects\_argmax_{w}F_{Conn}(w)$$

*F*_*conn*_ can be replaced with any other metric of bi-electrode connectivity. For example, the average-over-trials function could be replaced with the corresponding max or median function.

For each electrode, two initial connectivity maps are created and presented using matrices. The correlation connectivity map for electrode A is defined by

$$CM_{A}(B)=\frac{1}{N}\sum_{subject}F_{Conn}(A,B),$$

where N is the total number of subjects and W ∈ {50 ms, 100 ms, 150 ms, 200 ms}. Such a cross-correlation function with different possible window sizes alongside a small phase limit *φ* can be efficient in detecting synchronization of the flow of information in a system.

In a similar way, a standard deviation matrix map can be defined as follows:

*CM_STD*_*A*_(*B*) = *Std*_*subject*_*F*_*Conn*_(*A*,*B*). A healthy-schizophrenia patients Fisher-score matrix can then be created using the following equation:

$$FISHER(A,B)=\frac{\left|MEAN_{subject}HEALTHY_{CM_{A}(B)}-MEAN_{subject}SICK_{CM_{A}(B)}\right|}{\sqrt{{STD_{subject}HEALTHY\_CM\_STD_{A}(B)}^{2}+STD_{subject}{SICK\_CM\_STD_{A}(B)}^{2}}}.$$

Feature matrices are then created for additional features, for prediction and classification. Three characteristics were used to create these additional features: 1. Relative distances: the differences in the direct connectivity map are computed with respect to the reference electrode (see Fig 1). The relative distances are defined by

$$REL_{C}(A,B)=CM_{C}(A)-CM_{C}(B).$$

2. Feature multiplication: Multiplication elements that are extensions of the relative distances are defined. As reference electrode is shared by both elements of each multiplication matrix, these features amplify contribution of such successful, “multi-purpose” reference points. For each pair of relative elements, as defined above, a multiplication element is created, defined as follows:

$$\forall C,A_{1},A_{2},B_{1},B_{2}\in electrodes,MUL_{C}(A_{1},B_{1},A_{2},B_{2})=REL_{C}(A_{1},B_{1})REL_{C}(A_{2},B_{2})$$

3. Fisher relative score features matrix: This matrix is defined by

$$FISHER\_REL(A,B,C)=\frac{\left|MEAN_{subject}HEALTHY\_REL_{A}(B,C)-MEAN_{subject}SICK\_REL_{A}(B,C)\right|}{\sqrt{{STD_{subject}HEALTHY\_REL_{A}(B,C)}^{2}+STD_{subject}{SICK\_REL_{A}(B,C)}^{2}}}$$

The feature classification procedure collects only the most statistically significant discriminating features rather than the holistic network connectivity level. The procedure is as follows:

1. Set the parameters K_1_, K_2_ with the goal of limiting the number of electrodes involved in the process of interest. Construct a feature matrix F of size K_1_ electrodes x K_2_ features by letting each row *F*_*i*_ represent the source electrode i and its derived features. Steps 2–6 were tested with K_1_ chosen from {0.1N, 0.2N,.., N}, and K_2_ from {5%,10%}.
2. The elements of row i of the feature matrix are all features obtained from electrode i, as defined above: $CM_{i},STD_{CM_{i}},Fisher_{i},REL_{i},REL_{FISHER_{i}},MUL_{i}$.
3. To increase the clarity of the maps, only the top 10% of features in terms of healthy-schizophrenia patients variance were retained **[25]**.
4. For each row, calculate the average of the maximal K_1_ elements.
5. Choose the K_2_ features that have maximal scores. Use Laplacian regularization for feature scoring. Alternatively, another feature scoring method may also be used **[26]**.
6. Analyze the projected results for new segments of acquired data using a statistical model that features 2 states or models; compare the likelihood of each model (healthy individuals or schizophrenia patient) using Chi-squared tests followed by model comparison **[27]**. Classify the feature set according to the likelihoods of the two models.

Post-procedure validation: Perform cross-validation between patients’ data using the ‘leave one out’ method **[28]**. In each phase, one subject’s data is left out of the training set, and all properties are computed without using this data. Testing and predictions are then conducted with the data that was left out, treating this data as a fresh, newly acquired dataset.

### 2.2 –Experimental setup

We demonstrate an analysis of EEG data from a previously reported study **[24]**; this data provided evidence for contextual processing deficits in patients with schizophrenia by demonstrating alterations in the neural correlates of local contextual processing.

This experimental data was acquired while the subjects played a computer simulation game. The game featured a basic visual-stimuli identification-response task. Three different triangles were presented, each pointing in a different direction, with different patterns of recurrence. One of the shapes (‘P’ stimulus type) had a recurrence pattern that created a higher level of cognitive activity (“anticipation”) among the subjects in comparison to the other two; we focused on the EEG data that was recorded while responding to this specific stimulus. EEG data was recorded from a 64-electrode array using the ActiveTwo system (Biosemi, The Netherlands). External electrodes above and below the right eye monitored vertical eye movements, and electrodes placed laterally to the left and right eyes monitored horizontal eye movements. Signals were sampled at 512 Hz.

The stimuli consisted of black triangles on a gray background (triangles pointing to the left, upwards and to the right, in 90-degree increments). In each block, a total of 78 stimuli were presented for 150 ms each with an inter-stimulus interval (ISI) of 1 s. The stimuli blocks were a mixture of randomized sequences of standards and sequences including a three-standard predictive sequence. The predictive sequence always consisted of the three triangles facing to the left, up and to the right, always in that order. We focused our analysis on the response to the predictive sequences (“P” stimuli), as these are considered to be significant indicators of schizophrenia **[24]**.

In the preprocessing phase, a band-pass filter was implemented to pass frequencies in the interval of 0.1 Hz-30 Hz (excluding the gamma-1 and gamma-2 bands), and the filtered data were normalized by the standard deviation $nsig=\frac{sig}{std(sig)}$. The data were analyzed in 1.2-s epochs that began 200 ms prior to the appearance of the ‘P’ stimulus.

Eye blinks were removed using ICA **[29]**. Epochs containing misses (no button press 150–1150 ms post stimulus onset) and eye saccades were excluded from further analysis. EEG epochs with amplitudes of more than 75 μV at any electrode were excluded.

The parameters used for preprocessing and arguments for repetition analysis and optimization were the same as those used in a recent study that analyzed the same data set **[15]**.

The process described above was applied using a set of time windows separated into different frequency bands. Windows were created to overlap 90% of the 1.2-s epochs starting 200 ms before the stimulus and ending 1000 ms post stimulus. After pre-processing, bands of 0.1 Hz-30 Hz were separated into 6 equal 5-Hz intervals, and different combinations of these intervals (all subsets of the full band including the full band itself), in addition to the classical Alpha/Beta/Theta/Delta/Gamma bands, were tested. The responses to 5 to 15 stimuli from the beginning of each recording were analyzed, as described previously **[15]**; the number of responses analyzed was constant per iteration.

The full data set included 50 subjects: 25 were healthy, with no history of psychiatric illness or problematic behavior, and 25 had been diagnosed with schizophrenia, hospitalized and treated with appropriate medication. Signed, informed consent was obtained from all subjects. All participants had full capacity to consent, following a clinical evaluation of a psychiatrist. The local ethics committees of A Curuna University and Shaar Menashe Hospital approved the study. Patients were diagnosed with schizophrenia according to the Structured Clinical Interview for DMS-IV-Tr, and the severity of their symptoms was rated using the Positive (SAPS) and Negative (SANS) Syndrome Scale **[30]**. We note that during the recording phase, schizophrenia patients were receiving their regular medications.

## 3 Results

### 3.1 General overview

Our results are presented in four sections: (I) Significant connectivity maps with significant ability to discriminate between healthy subjects and schizophrenia patients. (II) Time windows that exhibit strong differences in connectivity features yet a high correlation in brain activity. (III) Fisher-based analysis to obtain connectivity maps calculated using the most significant connections. (IV) The accuracy of the discrimination methodology based on the prediction of schizophrenia.

### 3.2 Connectivity maps

The connectivity maps that were based on posterior electrodes showed large differences between healthy subjects and schizophrenia patients. The below figures demonstrate that from different posterior locations.

**Fig 3:**
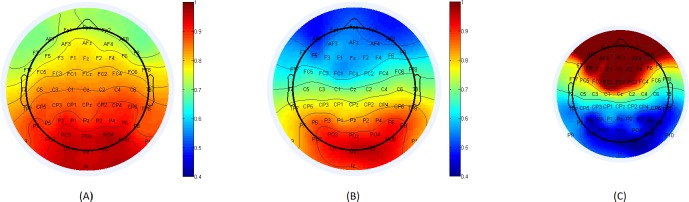
(A): Connectivity map for healthy subjects, with respect to reference electrode O2 located on the posterior area of the scalp. (B): Connectivity map of schizophrenia patients with respect to the same base electrode, O2. (C): Fisher score for the difference in each electrode’s connectivity with respect to reference electrode O2 between healthy subjects and schizophrenia patients. The location from which significant gaps in the flow of information between healthy subjects and schizophrenia patients originate (with respect to the specific reference electrode) can be determined.

Fig 3, below, depicts connectivity maps for healthy individuals and schizophrenia patients, with respect to reference electrode O2. The average score for electrode F2 among healthy subjects was 0.784 compared with 0.531 among schizophrenia patients. The average score gap between Cz and FCz was 0.122 among healthy subjects and 0.186 among schizophrenia patients.

**Fig 4:**
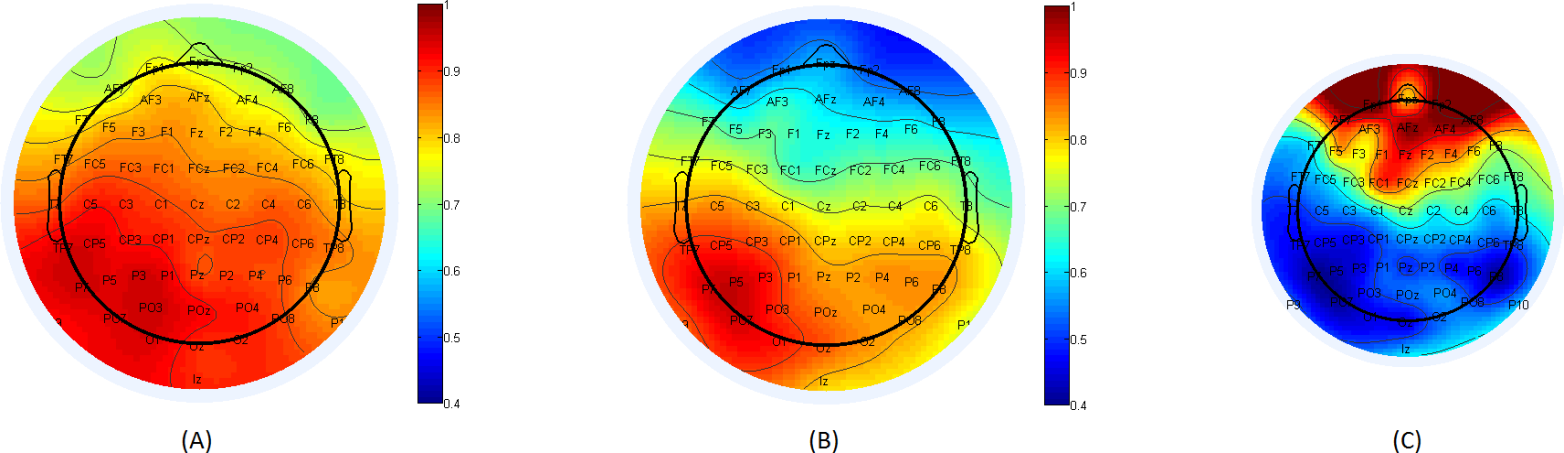
(A): Connectivity map for healthy subjects with respect to reference electrode P7, located in the posterior area of the scalp. (B): Connectivity map for schizophrenia patients with respect to the same base electrode, P7. (C): Fisher score for the difference in each electrode’s connectivity with respect to reference electrode, P7, between healthy subjects and schizophrenia patients. The location from which significant gaps in the flow of information between healthy subjects and schizophrenia patients (with respect to the specific reference electrode) can be determined.

Fig 4, below, shows connectivity maps of healthy and schizophrenia patients, with source electrode P7. The average score for electrode F2 among healthy subjects was 0.797 compared with 0.596 among schizophrenia patients. The average score gap between Cz and FCz was 0.083 among healthy subjects and 0.139 among schizophrenia patients.

**Fig 5:**
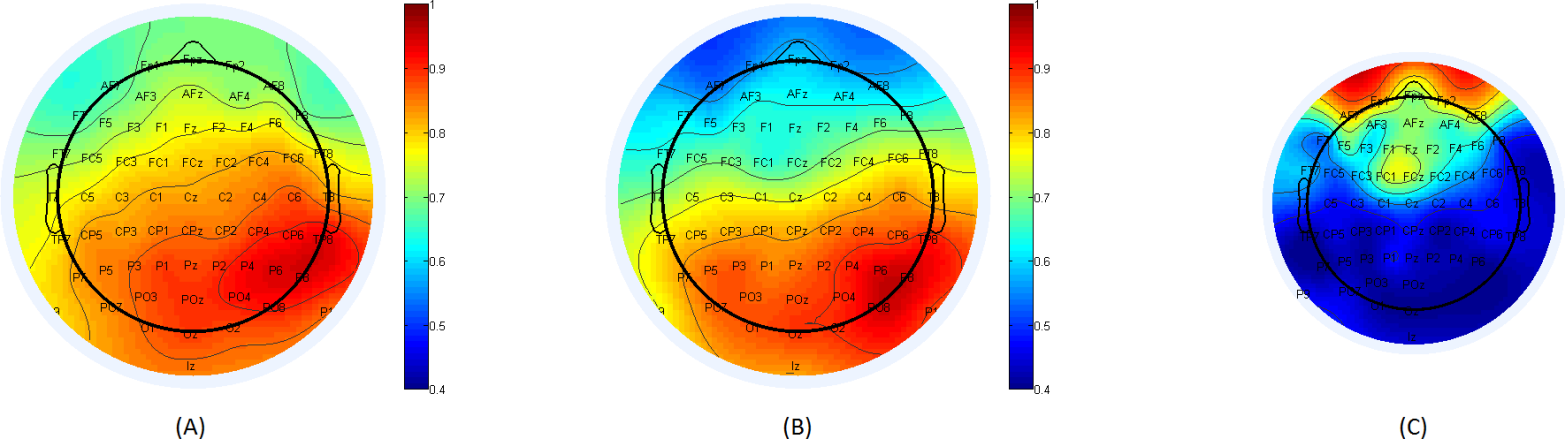
(A): Connectivity map for healthy subjects with respect to reference electrode P8 located on the posterior area of the scalp. (B): Connectivity map for schizophrenia patients with respect to the same base electrode, P8. (C): Fisher scores for the difference in each electrode’s connectivity with respect to the reference electrode, P8, between healthy subjects and schizophrenia patients. The location from which significant gaps in the flow of information between healthy individuals and schizophrenia patients (with respect to the specific reference electrode) can be determined.

Fig 5, below, shows connectivity maps for healthy and schizophrenia patients with source electrode P8. The average score for electrode F2 among healthy subjects was 0.76 compared with 0.642 among schizophrenia patients. The average score gap between Cz and FCz was 0.097 among healthy subjects and 0.14 among schizophrenia patients.

### 3.3 Time windows exhibit strong differences in connectivity yet a high correlation in brain activity

**Fig 6:**
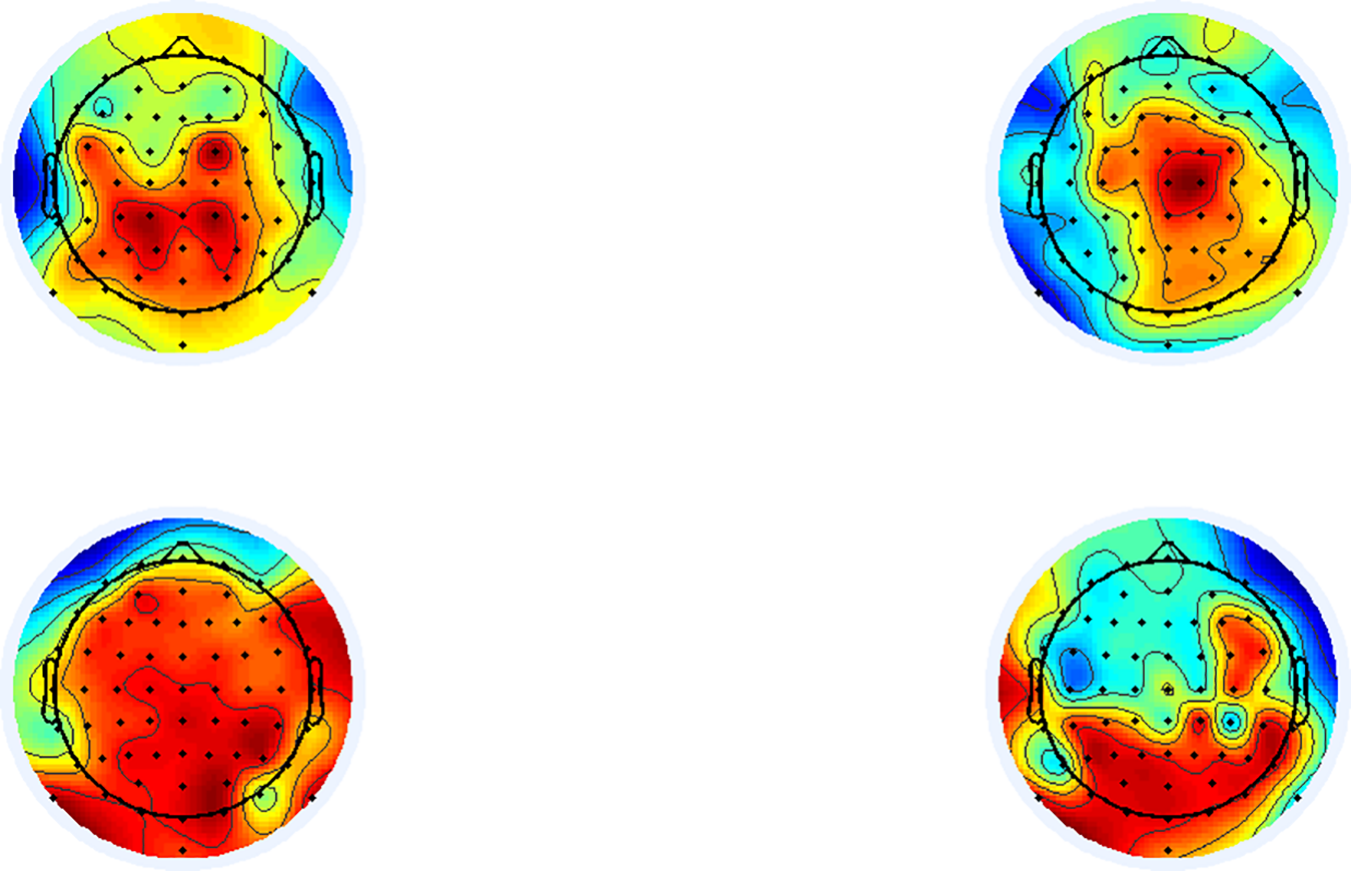
A (Top left): The brain activity of healthy subjects 420 ms after the presentation of a stimulus. Fig 6B (Top right): The brain activity of schizophrenia patients 420 ms after the presentation of a stimulus. Fig 6C (Bottom left): The connectivity features obtained 420 ms after the presentation of a stimulus for healthy subjects. Fig 6D (Bottom right): The connectivity features obtained 420 ms after the presentation of a stimulus for schizophrenia patients. Strong connectivity can be observed within the healthy subjects, and weak connectivity can be observed within the schizophrenia patients, even though the general brain activity level is similar in both populations.

Considering P9 as the source electrode and using a Euclidian distance function to find the closest activity states between patients and healthy individuals, a time window of 10 ms was found 420 ms after the appearance of the stimulus. Fig 6 below shows similar brain activity (in the top sub-plots) and relatively different connectivity distances (in the bottom sub-plots).

Considering the average of all base electrodes from the posterior section of the scalp, the average time at which brain activity was most similar was at 404 ms post stimulus. Among selected features by the methodology, the frequency 25 Hz - 30Hz was found most common (41%). Such activity in low-gamma band indicates neural oscillation is similar given substantial time interval from the stimulus.

### 3.4 Fisher-based analysis to identify the most significant connectivity maps

**Fig 7:**
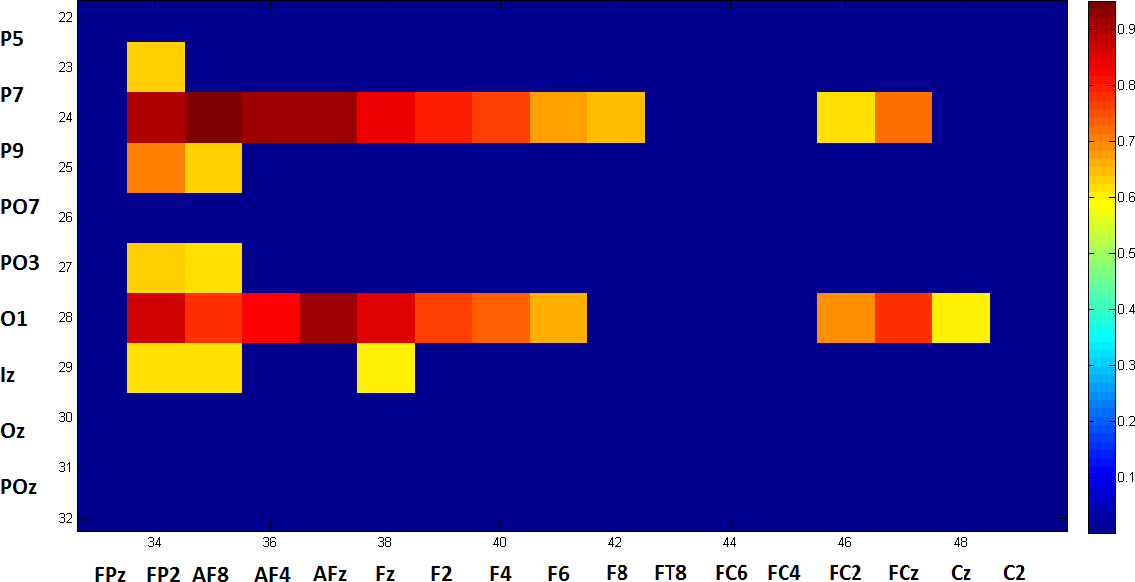
Connectivity maps showing electrode connectivity from posterior sources to frontal destinations with Fisher scores of at least >0.6, zoomed in to the relevant interval.

Considering Fisher score averages for connections based on posterior base electrodes, Fig 7, below, shows a map of connections with a Fisher score higher than a threshold of 0.6.

**Fig 8:**
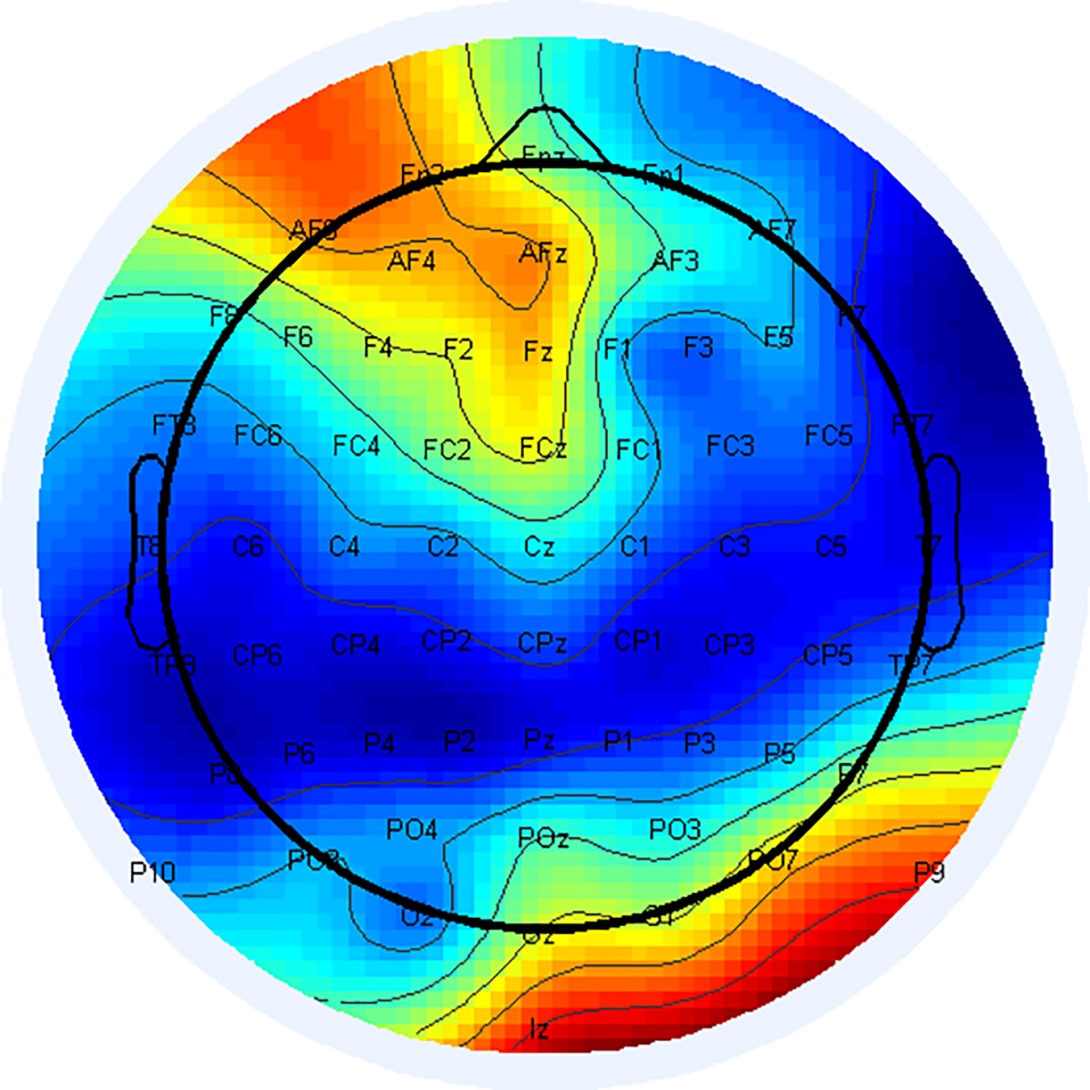
The Fisher score for each electrode based on the average score of the top five maximal connections in which it is involved. There is a significant fall in the aggregated Fisher score in the prefrontal area from 0.4 to 2.

After averaging the top 5 connection scores for each electrode, the following Fisher score connectivity map is obtained, as shown in Fig 8 below.

### 3.5 Accuracy of the prediction-based discrimination methodology

The accuracy of the connectivity maps in identifying schizophrenia was compared with that of several state-of-the-art methodologies for identifying this disorder. All methodologies were applied to the described dataset. The output of each methodology per tested data was binary and total correct calls as percentage were used as measurement for accuracy. The methods compared included the following:

1. TFFO: time and frequency optimization-based methodology for distinguishing between healthy individuals and schizophrenia patients based on input from several electrodes **[15]**.
2. P-300 derived features, as analyzed by “RT ANOVA”: a reaction times-based approach for discriminating between healthy individuals and patients diagnosed with schizophrenia **[24]**.
3. Latency-based methodologies: a basic comparison with subject latency analysis was performed by: (a) calculating for each stimulus its distance to peak energy amplitude and clustering using K-means (“PE-Latency”) **[30]**, (b) repeating the post feature extraction methodology but replacing the time-frequency features with the distribution of the energy peak located in the P300 area (“P300-RF-Latency”) **[31]**.
4. “Direct connectivity only” approach: using the “Connectivity Maps” methodology without relative features (or using only CM_VAL-based features, as presented in section 2), simulating an approach to evaluating the connectivity between two nodes with data only from the pair itself. Such an approach was used in a recently published patent **[21][22]**.

The strongest 5 connectivity maps were chosen according to maximum Fisher scores, as shown above in Fig 6). The connectivity maps’ features were stable and consistent during the cross validation phase.

Maximal separation was achieved using 11 stimuli from the beginning of each recording. The inclusion of all bands (use of a full set of bands) and use of the interval between 200 ms and 450 ms after the appearance of the stimulus helped to achieve the best result.

Using only 5 maximum-variance connections (involving a total of 5 electrodes) resulted in correct discrimination 92.1% of the time; this accuracy was very similar to that observed when using all connections (93.8%). In both cases, no false-positive cases with a strong statistical separation were observed (P = 0.0045).

No predictive classification results using this set of parameters contained false positives (classifying healthy subjects as schizophrenia patients).

## 4. Discussion

We have presented a novel method for quantifying the connectivity between brain regions as measured by the correlation of activity among electrodes. The novelty of this method is primarily due to two features of the method: i) a holistic view of the connectivity at every electrode is created, and this view is easily interpretable and summarizes the connectivity features, ii) a novel method is used to identify the initiating (reference) electrodes from which the connectivity map is most indicative of the difference between two pathologies. This method can be used to diagnose or monitor a pathology.

An analysis of the results obtained using the schizophrenia data suggests the following conclusions:

### I. Connectivity maps with a strong ability to discriminate between healthy subjects and schizophrenia patients

The results presented in section 3.2 show a repeating pattern of substantial differences at prefrontal electrodes when the base electrodes are at the posterior part of the brain. Figs 3–5 demonstrate clearly how the degradation of connectivity strength is being accelerated within the patients. Target electrode F2 brings an absolute score difference that ranges from 18.74% to 34.5% depending on the posterior reference electrode chosen.

Links in the prefrontal area also show significant differences in connectivity scores based on the selection of the posterior base electrodes. Specifically, the link Cz-FCz degradation gap between healthy subjects and schizophrenia patients ranges from 35.8% to 67.5%, with maximum results obtained with P7 as the base electrode.

These results indicate that information relay changes in an abnormal manner primarily in the prefrontal area. These results correlate with past connectivity analyses conducted using fMRI **[32][33]**. A high difference in the score of target electrode F2 has been found in previous work using the TFFO method **[15]**; in that study, F2 was found to be able to discriminate between healthy subjects and schizophrenia patients better than any other single electrode.

Frontal electrodes have previously been shown to be significant in recent research that addressed schizophrenia **[24][34]**, together with other illnesses with psychiatric components, such as Parkinson’s disease **[32]**.

The results in both scenarios—using all the Connectivity Maps and using those from picked 5 electrodes—featured no cases of false-positives. This is an indication that the Connectivity Maps’ features are powerful for discriminating between the healthy subjects and the Schizophrenia patients with less disease severity, that were the main source of false-positives in previous work **[15][24][31]**. It shall be also noted that while “Leave One Out” is a standard cross-validation technique, it is known to be relatively optimistic **[35]** and more likely statistically to converge with lower false positives, especially when analyzing relatively small data sets.

### II. Time windows in which a substantial difference in connectivity is observed yet in which brain activity is highly correlated

As presented in section 3.3, connectivity features were not necessarily correlated with brain activity. Between 400 and 425 ms post stimulus, when brain activity was most similar between healthy individuals and schizophrenia patients, connectivity features differed significantly.

Maximal classification accuracy was obtained in the time window of 200 ms-450 ms, suggesting that the P300 area is most able to discriminate between patients and health individuals, as was suggested previously **[33]**. There is also a ‘latency recovery’ effect, in which schizophrenia patients’ response times to a stimulus improved during the recording when the stimulus was followed by a visual analysis task. Such an effect is evident in a number of psychiatric illnesses **[33][36]**.

Our connectivity maps features demonstrated strong connectivity in both the posterior and the prefrontal/frontal areas within healthy subjects, while strong connectivity was only observed in the posterior area within the patients with schizophrenia. As this observation is consistent with previous findings of posterior-frontal differences **[24]**, it provides clear evidence of the independence of the features obtained in relation to more common brain activity measurements used as input for feature classification. Methodology results demonstrated significantly superior results to the standard latency-based methodologies **[30][31]**.

### III. Fisher-based analysis to obtain connectivity maps using the most significant connections

In the results in section 3.4, one can see that the most significant results in the aggregated Fisher score matrix (as presented in Fig 8) involve the posterior electrodes and the frontal electrodes.

Delving into the data more deeply (such as in the zoomed area shown in Fig 7) reveals that the most significant connections are long-distance ones (connections between posterior electrodes and frontal electrodes, instead of close connections). Fig 8 also reveals that the smallest number of electrodes with such significant connections can be observed in the central/central parietal area, between Cz and CPz, for example.

There are two implications to these findings. First, they support the claim that measuring the connectivity strength between the most posterior electrode and the frontal electrode is a strong tool for classifying and predicting schizophrenia.

Secondly, these findings help us to understand that although the maximal differences are observed for the longest distances, the bigger picture is that an examination of degrading connectivity features in schizophrenia patients shows us that changes in the flow of information occur in their most significant form in prefrontal areas.

### IV. The accuracy of the discrimination methodology based on its ability to predict schizophrenia

As observed in the results presented in Table 1, it is evident that connectivity maps were able to discriminate between healthy subjects and schizophrenia patients. As the data used for this analysis was acquired from each subject in only the first minute of testing (the optimal number of repetitions from the beginning of the recording was in the range of 8–12, depending on the set of electrodes used), these results can be considered to be firm. The best analysis configuration used all bands of frequencies (up to 30Hz as filtered in the preprocessing phase); using all frequency bands produced better results than using only a subset of the frequencies.

**Table 1:** Discrimination accuracy for each tested methodology.

Methodology	Discrimination Accuracy	Specificity Rate	Sensitivity Rate	Significance P-Value
Connectivity Maps (All Maps)	93.8%±4%	100%	87.6%±5.1%	0.0041
Connectivity Maps (Best 5)	92.4%±3.8%	100%	84.8%±4.7%	0.005
TFFO **[15]**	88.7%±4%	100%	77.4%±5%	0.0078
Connectivity Maps—Direct Connectivity Only **[21][22]**	88.7%±5.5%	96.5%±2.5%	81%±5.8%	0.0089
P300-Derived Features–Reaction Time **[24]**	73.9%±4.3%	87.3%±3.1%	60.6%±4.6%	0.043
P300-RF-Latency **[31]**	68.1%±4.8%	82%±3.9%	54.2%±6.9%	0.0919
PE-Latency **[30]**	64.5%±5.5%	74.2%±4.2%	54.8%±6.4%	0.1746

These results are clearly powerful in comparison with standard ANOVA methodologies **[24]** and latency-based feature analyses **[30]**. There is also a significant improvement compared to optimization-based methodologies with a strong machine-learning focus **[15]**.

Results of section 3.5 regarding the use of 5 electrodes to obtain classification accuracy just shy to the one achieved when using all the data, reaffirm results of past research **[15]** of ability to achieve strong discrimination using a small subset of the original input electrodes.

Using direct connectivity only and achieving significantly less accurate classification results in comparison with the full list of features clearly indicates the orthogonal added value of the three-electrode approach, in which the connectivity between a pair of electrodes is measured with respect to a third electrode rather using input from that pair alone. Approaches considering only direct connectivity and related differences have been described previously. **[21][22]**

Results also indicate that robust discrimination can be achieved with an analysis of about a minute of playing a certain game with a strategically placed set of only 4–6 electrodes. The relatively short time needed to acquire data for this analysis can be an enabling feature as patients with psychiatric disorders have difficulty completing tasks as recording sessions get longer.

## 5 Conclusion and further work

We have presented a novel tool for the analysis of connectivity termed ‘Connectivity Maps’. We have demonstrated that features identified using these maps, which were acquired using only a small number of strategically-placed electrodes and a relatively short length of recording time, can predict and classify schizophrenia using EEG data with high accuracy.

We are aware that the results we have presented may merely be distinguishing between subjects who are taking anti-schizophrenia medications and those who are not. Further studies should determine whether our findings are more correlated with the schizophrenia condition or schizophrenia medications.

The described method is relatively efficient as the total number of created maps is *n*^3^. In case number of nodes is high, further efficiency can be gained by adding a distance function D between nodes and limiting features computed only to nodes that meet a distance threshold from the relevant reference node. Such modification can dramatically reduce the size of the connectivity maps.

The method we describe here is very suitable for describing changes in connectivity between groups of subjects. Unlike other connectivity methods, this method also suggests the physical location (or the electrode closest to it) at which observed changes in connectivity are best exemplified.

Because results using this method can be easily interpreted, they can aid research into the origin of schizophrenia and serve as a good diagnostic tool. This method can also be used to provide neurofeedback. Portable, fast and inexpensive EEG, together with such effective analysis tools, can therefore result in new diagnostic and treatment alternatives, especially for mobile health, which is used to verify the effectiveness of medication, allowing real-time treatment decision-making via medicine pumps, among other possibilities. All of these benefits can be applicable to other pathological conditions, as well, including disorders affecting emotions and attention, as the Connectivity Maps method is very general.

Embedded machine learning mechanisms can help devices to assist or even replace traditional medical and diagnostic procedures in the future, as accuracy and the methods for analyzing the severity of psychiatric disorders may be improved to the point where their credibility surpasses that of existing approaches. It may be possible to use this method for brain-computer interface applications as well.
